# Supplementary material for: Global mapping of RNA-chromatin contacts reveals a proximity-dominated connectivity model for ncRNA-gene interactions
Source: Nat Commun. 2023 Sep 28;14:6073. doi: 10.1038/s41467-023-41848-9 (PMC10539311; doi:10.1038/s41467-023-41848-9)
Supplement: Supplementary file 3 — Description of Additional Supplementary Files [file 41467_2023_41848_MOESM3_ESM.pdf]

Description of Additional Supplementary Files

File Name: Supplementary Data 1
Description: **Differential Expression by total RNA-seq and in the caRNA transcriptome by ChARseq, related to Figure 1.** Log2 Fold Change, p-values, and False Discovery Rate adjusted p-values for differential expression in DE vs ES cells of Gencode genes (separating reads from exons and introns), and either including (first tab) or excluding UTLs (second tab) computed using DESeq2. DESeq2 was run independently on RNA-seq count matrices (4 ES and 4 DE biological replicates) for total RNA-seq, and on ChAR-seq count matrices (2 ES and 2 DE biological replicates) for the caRNA transcriptome. Genes with fewer than 10 counts across samples were removed from analysis. All DESeq2 parameters were set to their default value, except for the sample depth normalization step which was done using only counts from exonic reads as standard in RNA-seq analysis.

File Name: Supplementary Data 2
Description: **Chromatin Association scores, related to Figure 1.** Chromatin association scores for exons and introns of Gencode genes and UTLs. All scores, log 2 fold change and p-values were computed as described in Methods.

File Name: Supplementary Data 3
Description: **Catalog of UTLs and their classification, related to Figure 2.** List of Unannotated Transcribed Loci and their classifications.

File Name: Supplementary Data 4
Description: **Final transand cis-delocalization scores averaged over replicates, related to Figure 4.** Scores were first calculated independently in 2 biological replicates of ES cells and 2 biological replicates DE cells, then averaged per cell type. Pre-averaged scores are given in Supplementary Data 8-9. All details on the scores and p-values computations are given in Methods and Supplementary Note 2.

File Name: Supplementary Data 5
Description: **Catalog of RNAs with extreme delocalization scores, related to Figure 4.** RNAs with a positive cis- or transdelocalization score and adjusted p- value (p.high.corr) < 0.05 for having a more extreme delocalization score than an mRNA are shown in the “delocalized_cis” and “delocalized_trans” tab, respectively. RNAs with a negative cis- or transdelocalization score and adjusted pvalue (p.low.corr) < 0.05 for having a more extreme delocalization score than an mRNA are shown in the “ultralocalized-cis” and “ultralocalized_trans” tab, respectively. All details on the scores and p-values computations are given in Methods and Supplementary Note 2.

File Name: Supplementary Data 6
Description: **List of RNADNA contacts not predicted by the generative model, related to Figure 5.** Contacts are defined using 100 kb bins on the DNA side, and individual RNAs (annotated exon, intron, or UTL) on the RNA side. Contacts not predicted by the model were those with a Log2 Fold Change observed over model greater than 1.3 and an adjusted p-value less than 0.05, as in Figure 5c,e.

File Name: Supplementary Data 7
Description: **Coarse graining of Gencode V29 annotations.** Coarse grained annotations for Gencode V29 data types.

File Name: Supplementary Data 8

Description: **transdelocalization scores by sample.** Scores for individual replicates used for averaging in Supplementary Table4.

File Name: Supplementary Data 9
Description: **cisdelocalization scores by sample.** Scores for individual replicates used for averaging in Supplementary Table4.

File Name: Supplementary Data 10
Description: **Oligonucleotide sequences.** List of oligonucleotide primers used in this study.
