# Supplementary material for: Global mapping of RNA-chromatin contacts reveals a proximity-dominated connectivity model for ncRNA-gene interactions
Source: Nat Commun. 2023 Sep 28;14:6073. doi: 10.1038/s41467-023-41848-9 (PMC10539311; doi:10.1038/s41467-023-41848-9)
Supplement: Supplementary file 14 — Reporting Summary [file 41467_2023_41848_MOESM14_ESM.pdf]

Corresponding author(s): Aaron F. Straight
Last updated by author(s): Aug 29, 2023

Reporting Summary

Nature Portfolio wishes to improve the reproducibility of the work that we publish. This form provides structure for consistency and transparency in reporting. For further information on Nature Portfolio policies, see our Editorial Policies and the Editorial Policy Checklist.

Statistics

For all statistical analyses, confirm that the following items are present in the figure legend, table legend, main text, or Methods section.

n/a	Confirmed	
☐	☒	The exact sample size (*n*) for each experimental group/condition, given as a discrete number and unit of measurement
☐	☒	A statement on whether measurements were taken from distinct samples or whether the same sample was measured repeatedly
☐	☒	The statistical test(s) used AND whether they are one- or two-sided<br>*Only common tests should be described solely by name; describe more complex techniques in the Methods section.*
☐	☒	A description of all covariates tested
☐	☒	A description of any assumptions or corrections, such as tests of normality and adjustment for multiple comparisons
☐	☒	A full description of the statistical parameters including central tendency (e.g. means) or other basic estimates (e.g. regression coefficient) AND variation (e.g. standard deviation) or associated estimates of uncertainty (e.g. confidence intervals)
☐	☒	For null hypothesis testing, the test statistic (e.g. *F*, *t*, *r*) with confidence intervals, effect sizes, degrees of freedom and *P* value noted<br>*Give P values as exact values whenever suitable.*
☐	☒	For Bayesian analysis, information on the choice of priors and Markov chain Monte Carlo settings
☐	☒	For hierarchical and complex designs, identification of the appropriate level for tests and full reporting of outcomes
☐	☒	Estimates of effect sizes (e.g. Cohen's *d*, Pearson's *r*), indicating how they were calculated

Our web collection on statistics for biologists contains articles on many of the points above.

Software and code

Policy information about availability of computer code

Data collection	Cell Profiler (3.1.8) was used for obtaining maximum intensity projection of microscopy images
Data analysis	All analysis tools used are listed in the text. Custom analysis code was deposited on github: https://github.com/straightlab/charseq-pipelines, https://github.com/straightlab/chartools, https://github.com/straightlab/tagtools

For manuscripts utilizing custom algorithms or software that are central to the research but not yet described in published literature, software must be made available to editors and reviewers. We strongly encourage code deposition in a community repository (e.g. GitHub). See the Nature Portfolio guidelines for submitting code & software for further information.

Data

Policy information about availability of data

All manuscripts must include a data availability statement. This statement should provide the following information, where applicable:

- Accession codes, unique identifiers, or web links for publicly available datasets
- A description of any restrictions on data availability
- For clinical datasets or third party data, please ensure that the statement adheres to our policy

A data availability statement has been added to the manuscript and all data is publicly accessible as GEO accession GSE240435 and in the github repositories https://github.com/straightlab/charseq-pipelines, https://github.com/straightlab/chartools, https://github.com/straightlab/tagtools

Research involving human participants, their data, or biological material

Policy information about studies with human participants or human data. See also policy information about sex, gender (identity/presentation), and sexual orientation and race, ethnicity and racism.

Reporting on sex and gender	N/A
Reporting on race, ethnicity, or other socially relevant groupings	N/A
Population characteristics	N/A
Recruitment	N/A
Ethics oversight	N/A

Note that full information on the approval of the study protocol must also be provided in the manuscript.

Field-specific reporting

Please select the one below that is the best fit for your research. If you are not sure, read the appropriate sections before making your selection.

[x] Life sciences [ ] Behavioural & social sciences [ ] Ecological, evolutionary & environmental sciences

For a reference copy of the document with all sections, see nature.com/documents/nr-reporting-summary-flat.pdf

Life sciences study design

All studies must disclose on these points even when the disclosure is negative.

Sample size	Balanced number of replicates were used across cell lines. For ChAR-seq, we used 2 replicates per cell line, as standard for assays requiring ultra deep sequencing (e.g. HiC, etc.).
Data exclusions	All exclusion rules were described in the text. For ChAR-seq differential maps, RNA-DNA contacts with fewer than a preset number of reads (less that 2 samples with at least 10 reads) were removed. This follows standard recommendations for prefiltering in differential RNA-seq (e.g. DESeq2), to limit the number of hypothesis tested.
Replication	Replication was assessed by comparing inter-replicates vs intra-replicate correlations.
Randomization	Not applicable. The data in the manuscript compared two cell states (ES vs DE) with appropriate controls. Randomization would not have been appropriate for the data type.
Blinding	All tests were done through automated computational routines and did not involve any form of manual scoring. For experiments, the only groups were cell lines pre- and post- differentiation and those have to be known by the experimenter to apply cell specific protocols thus would not be appropriate for blinding.

Reporting for specific materials, systems and methods

We require information from authors about some types of materials, experimental systems and methods used in many studies. Here, indicate whether each material, system or method listed is relevant to your study. If you are not sure if a list item applies to your research, read the appropriate section before selecting a response.

Materials & experimental systems

n/a	Involved in the study	
[ ]	[x]	Antibodies
[ ]	[x]	Eukaryotic cell lines
[x]	[ ]	Palaeontology and archaeology
[x]	[ ]	Animals and other organisms
[x]	[ ]	Clinical data
[x]	[ ]	Dual use research of concern
[x]	[ ]	Plants

Methods

n/a	Involved in the study	
[x]	[ ]	ChIP-seq
[x]	[ ]	Flow cytometry
[x]	[ ]	MRI-based neuroimaging

Antibodies

Antibodies used	Goat anti-Sox17, R&D Systems, AF1924

	Rabbit anti-Nanog, Bethyl Labs, A300-397A<br>Rabbit anti-Sox2, Cell Signaling, 3579T<br>Rabbit anti-FoxA2, EMD Millipore, 07-633<br>Goat anti-Rabbit Alexa-647, Thermo-Fisher, A32733<br>Donkey anti-Goat Alexa-568, Thermo-Fisher, A11057
Validation	Validation information for antibodies was performed by the manufacturers

Eukaryotic cell lines

Policy information about cell lines and Sex and Gender in Research

Cell line source(s)	Human H9 embryonic stem cells, WiCell, WA09
Authentication	Authentication performed by WiCell
Mycoplasma contamination	Mycoplasma testing and characterization performed by WiCell
Commonly misidentified lines<br>(See ICLAC register)	*Name any commonly misidentified cell lines used in the study and provide a rationale for their use.*
